# Spatial Differences in Avoidable Mortality Across 581 European Districts, 2002–2019

**DOI:** 10.1007/s10680-025-09761-7

**Published:** 2025-12-09

**Authors:** Sophie Stroisch, Michael Mühlichen, Pavel Grigoriev, Tobias Vogt

**Affiliations:** 1 Population Research Centre, Faculty of Spatial Sciences, University of Groningen, Groningen, The Netherlands; 2 Institute of Social Sciences, Carl von Ossietzky University of Oldenburg, Oldenburg, Germany; 3 Federal Institute for Population Research, Wiesbaden, Germany; 4 Prasanna School of Public Health, Manipal Academy of Higher Education, Manipal, Karnataka India

**Keywords:** European Union, Avoidable mortality, Regional inequalities, Hot-spot-analysis, Cross-border

## Abstract

Despite ongoing efforts to reduce health disparities, substantial mortality differences persist across and within European countries. Avoidable mortality, i.e. deaths preventable through timely medical care or effective public health measures, provides a useful framework for assessing these inequalities. While previous studies largely focus on national differences, this study examines spatial differences and trends in avoidable mortality at the district level. We analysed official cause-of-death statistics for 581 districts across 10 European countries from 2002 to 2019, using age-standardised death rates (SDR) as the primary outcome. Spatiotemporal clusters were identified using emerging hotspot analysis. Our results show that SDRs for avoidable mortality declined across most districts for both sexes, yet marked disparities persist between men and women and between amenable and preventable mortality. Districts in Central and Eastern Europe consistently exhibited higher SDRs than those in Western and Southern Europe, while Germany occupies an intermediate position with particularly high rates for amenable mortality. Spatiotemporal hotspot analysis confirmed these patterns and showed that persistent high-mortality (hotspots) and low-mortality (coldspots) clusters persist, reflecting both substantial within-country inequalities and cohesive cross-border mortality patterns. These findings suggest that national boundaries are not always the most meaningful lines for explaining health outcomes. Instead, local socioeconomic and structural determinants of health play a central role. Persistent hotspots highlight areas where targeted interventions are urgently needed, while coldspots may offer lessons for best practices. Addressing these inequalities requires targeted, place-based, and transnational strategies to effectively reduce health inequalities in Europe.

**Supplementary Information:**

The online version contains supplementary material available at 10.1007/s10680-025-09761-7.

## Background

### Mortality Differences in Europe

While significant progress has been made in improving population health, overall mortality outcomes and life expectancy continue to differ substantially across European countries (Hrzic et al., 2021; Santos et al., 2020; Sauerberg et al., 2024). Historically, Western and Northern European countries have acted as vanguards in mortality reduction, experiencing earlier and more sustained improvements in life expectancy. In contrast, many Eastern and some Southern European regions have functioned as laggards, with slower and more uneven progress (Vallin & Meslé, 2004). These dynamics have resulted in persistent East–West mortality gaps. Despite some signs of convergence, the mortality divide between vanguard and laggard regions has proven resilient over time (Hrzic et al., 2021; Sauerberg et al., 2024). This pattern highlights the ongoing challenge of achieving equal health standards across the European Union (EU).

Several structural, demographic, and behavioural factors underpin these disparities. The causes of death, such as cardiovascular diseases, cancers, respiratory illnesses, and external causes, vary significantly from one country to another. For example, cardiovascular diseases, particularly ischemic heart diseases, remain a leading cause of mortality in Eastern Europe and continue to account for a substantial portion of the mortality gap compared to Western countries (Hammond-Haley et al., 2021; Meslé & Vallin, 2017; Townsend et al., 2016; Wéber et al., 2023). Moreover, differences in mortality are influenced by broader health determinants, including socioeconomic conditions, education, and environmental exposure (Rahman et al., 2022; Richardson et al., 2014; Tetzlaff et al., 2024; Zazueta-Borboa et al., 2023). Health-related behaviours, such as smoking and alcohol consumption, also play a significant role in shaping outcomes associated with preventable mortality. Eastern European nations, in particular, contend with a heavy burden of behavioural risk factors, which contribute to both lower life expectancy and higher premature mortality, further exacerbating the persistent East–West divide (Grigoriev et al., 2020; Janssen, 2021; Trias-Llimós et al., 2018).

### Avoidable Mortality to Measure Health Care Effectiveness

A key determinant underlying these mortality differences is the performance of healthcare systems and public health policies. To better understand their impact, scholars and policymakers make use of the concept of avoidable mortality, a metric that captures deaths which could potentially be prevented through effective healthcare delivery or public health interventions (Nolte & McKee, 2004; Page et al., 2006; Rutstein et al., 1976). It comprises two key components: amenable mortality and preventable mortality. Amenable mortality refers to deaths that should not occur if individuals receive timely and appropriate medical care. Examples include deaths from treatable conditions such as infectious or early-stage cancers. In contrast, preventable mortality encompasses deaths that could be avoided primarily through public health measures, behavioural changes, or policy interventions, rather than clinical treatment. Examples include deaths due to smoking-related illnesses and fatalities from traffic accidents. Conversely, deaths not classified as avoidable arise from conditions that are currently considered neither preventable nor amenable to treatment. These include, for example, neoplasms with poor prognoses and limited prevention or treatment options, such as pancreatic or ovarian cancer, as well as neurodegenerative diseases (e.g. Alzheimer’s disease), severe congenital anomalies, and certain rare, aggressive disorders. Deaths occurring at advanced ages (≥ 75 years) are also excluded by definition, as both the ‘avoidability’ of death and the reliability of death certification become increasingly uncertain in this age group (Nolte & McKee, 2004, p.65).

Scholars have analysed trends in avoidable mortality across the European Union, documenting significant overall reductions while simultaneously revealing persistent inequalities (Cherla et al., 2024; Hrzic & Vogt, 2024; Jarčuška et al., 2017; Mackenbach et al., 2015). These studies illustrate that these disparities often mirror broader socio-economic and regional divides. Western European countries consistently demonstrate lower avoidable mortality rates compared to their Eastern European counterparts. Specific causes of death, such as cardiovascular diseases, particularly ischemic heart disease, have shown some of the most substantial reductions in avoidable mortality over recent decades. Nevertheless, ischemic heart disease remains the leading cause of avoidable deaths in several underperforming regions (Cherla et al., 2024). These patterns reflect not only uneven advancements in healthcare quality and accessibility but also significant gaps in the effectiveness of public health and preventive initiatives across Europe.

### The EU Context

The reduction of health disparities is a central tenet of the EU’s commitment to harmonise living standards. Recognising the value of avoidable mortality as an indicator, the EU and its agencies use it as a key metric to evaluate the performance and equity of national health systems and to monitor progress toward health convergence (OECD & Eurostat, 2022). This focus is embedded within broader cohesion policies and structural funds, such as the European Regional Development Fund, which supports less developed regions through investments in infrastructure, and services. Another initiative by the EU is the promotion of mutual learning through the Open Method of Coordination, a voluntary framework that encourages member states to share best practices and adopt successful policies from one another. Nevertheless, considerable variation remains in how national healthcare systems are structured and financed across the EU, as healthcare remains under national jurisdiction. For example, nations such as Spain and Italy exemplify government-funded healthcare systems, wherein the provision of care is predominantly public. In contrast, Austria and Germany adopt a social insurance model that incorporates both public and private healthcare providers (Reibling, 2010). Furthermore, a stark contrast in healthcare spending persists between Eastern and Western Europe, with Western nations allocating over 10% of their GDP to healthcare, while some Eastern countries allocate only 6% to 7% (Eurostat, 2024). Nevertheless, although this spending might be linked to improvements in life expectancy (Vogt & Kluge, 2015), there is also evidence to suggest that increased spending does not invariably lead to particularly high levels of life expectancy (Jasilionis et al., 2023). Taken together, these contrasts suggest that national-level comparisons provide only a partial picture of health inequalities across the EU.

### The Need for a Regional Scope

To capture the dynamics hidden behind national averages, it is necessary to extend the analysis of avoidable mortality to the regional level. Examining health outcomes at the regional level, rather than solely at the national level, allows for a more nuanced understanding of disparities shaped by local healthcare accessibility, cross-border interactions, and regional socio-economic conditions. Despite its importance, comparisons at the sub-national level across European countries remain rare. Recent studies have begun to investigate mortality patterns at provincial and district levels, offering important insights that are often obscured by national averages (see e.g., Bonnet et al., 2024b; Hrzic et al., 2021; Richardson et al., 2014; Sauerberg et al., 2024).

However, when focusing specifically on avoidable causes of death, the sub-national and cross-national evidence base is even more limited. Research in this area has typically been confined to either a specific geographic scope, such as the German-speaking regions of Europe (Mühlichen et al., 2023), or to particular causes, like alcohol-related deaths in Eastern European districts (Grigoriev et al., 2016, 2020) or cancer survival rates in the German-Danish border region (Rudolph et al., 2023; Storm et al., 2015). Consequently, a comprehensive, large-scale assessment of overall avoidable mortality at a fine geographical level across diverse European contexts is still missing. This gap is particularly evident in cross-border regions, where the interplay of different national health policies creates unique conditions that have received limited systematic attention (Stroisch et al., 2025a). Therefore, our study addresses this need by providing the first large-scale, spatiotemporal analysis of avoidable mortality across a wide range of European regions.

## Research Objective

This study seeks to extend the existing knowledge by investigating spatial differences and their changes over time in avoidable mortality at a European district level, contributing to the discourse on regional mortality disparities in Europe that goes beyond national borders. To achieve this, our objectives are two-fold. First, we map and describe the spatial trends in amenable and preventable mortality across all districts of 10 EU member states during the last two decades before the COVID-19 pandemic. Second, we employ spatio-temporal analysis to identify significant clusters of high-mortality (hotspots) and low-mortality (coldspots), which uncovers the underlying geography of health inequality, pinpointing how mortality clusters are organised spatially, both within countries and across national borders.

## Data and Methods

### Data

Before conducting our analyses, we harmonised population and death count data collected from statistical authorities across ten EU member states: Austria, Belgium, Czechia, France, Germany, Italy, Poland, Slovakia, Spain, and Switzerland. These data are stratified by year, country, district, cause of death, sex, and age group. Causes of death are categorised using the ninth and tenth revisions of the International Classification of Diseases (ICD), depending on the country and the year. The dataset includes 581 districts, of which 205 are border districts. These border regions are classified according to Eurostat’s Methodological Manual on Territorial Typologies (European Commission, 2019), which explicitly defines border regions at the third level of the Nomenclature of Territorial Units for Statistics (NUTS-3). To capture these dynamics, our analysis therefore required data to be scaled to at least the NUTS-3 level. At higher levels of aggregation, e.g., NUTS-2 level, border regions can no longer be consistently identified, making the district-level scale essential for the study’s objectives. As a result, we had to exclude some EU member states due to the lack of available data.

To ensure consistency across countries, we rely on data at the NUTS-3 level for Belgium, Czechia, Italy, Poland, Spain, Slovakia, and Switzerland, or on an equivalent administrative unit for Austria (*Bezirke*), and Germany (*Raumordnungsregionen*). For Italy, NUTS-3 regions have been harmonised to account for territorial changes over time. Throughout this paper, we use the terms *district* and *region* to distinguish between different spatial scales. The term *district* refers to the individual administrative units used in our dataset. In contrast, a *region* refers to a larger area encompassing several districts that can spread across borders, such as the Alpine region or a cross-border region shared between two countries.

Computing mortality rates at such a granular geographical level occasionally yields unstable measures. Death counts in less-populated districts can often be very low or zero, particularly when it comes to specific causes of death. To overcome this issue, we pooled the data into three-year periods, starting from 2002 to 2004, which was primarily before EU enlargement, and continuing up to 2017–2019, just prior to the COVID-19 pandemic. The exclusion of the pandemic years was deliberate. At the time of writing, cause-specific mortality data were available only up to 2021, which, together with 2020, corresponds to the peak of COVID-19 mortality (World Health Organization, 2025). Including these years would not only be incoherent with the three-year observation periods used in this study but would also, and more critically, risk overshadowing the long-term dynamics we aim to capture by conflating past trends with the exceptional mortality of the pandemic. Moreover, previous research has shown that the geographic distribution of COVID-19 mortality shifted substantially between 2020 and 2021, from initial hotspots in Northern Italy and Central Spain to Eastern European regions with lower pre-pandemic life expectancy (Bonnet et al., 2024a).

### Avoidable Mortality Classification

For our analysis, we are following the classification by Mühlichen et al. (2023), which was developed based on earlier works from Nolte and McKee (2004, 2012) and Page et al. (2006). In contrast to the classification developed recently by OECD/Eurostat (2022), it allows the identification of amenable and preventable causes of death based on three-digit ICD codes, which are more readily available and less susceptible to bias due to variations in coding accuracy on the fourth digit. Furthermore, it is not only compatible with ICD-10 but also with the two preceding versions (ICD-8 and ICD-9), making it more suitable for long-term analyses. The full list of amenable and preventable causes of death is provided as Online Resource 1.

### Analytical Strategy

We address our research questions in two steps. First, to investigate trends in avoidable mortality, we computed and mapped age-standardised death rates across all 581 districts. Second, to identify spatiotemporal clusters of high or low avoidable mortality over time, we applied an emerging hotspot analysis.

#### Mapping Avoidable Mortality Differences

Since the age composition of a population heavily influences mortality rates, we calculated age-standardised death rates (SDR) using the direct method of standardisation. After combining all amenable and preventable causes into two groups, we applied the age-specific mortality rates for each district and sex to a common reference, the European standard population of 2013 (Pace et al., 2013).

We present the results using deciles, categorising the SDR values into ten equal groups for both time periods, 2002–2004 and 2017–2019, as well as for amenable and preventable mortality combined. We did this for men and women, separately. This approach allows us to highlight both spatial and temporal differences in avoidable mortality. To complement the maps, we also provided boxplots and interquartile ranges (IQR) showing the distribution of SDRs across districts, stratified by country, and their changes over time. The additional plots can be found in the Online Resource 3.

#### Emerging Hotspot Analysis

The computed SDRs served as the input for the Emerging Hotspot Analysis. This method combines spatial and temporal statistics to classify how mortality clusters evolve over time. Briefly, it first uses the Getis-Ord Gi* statistic (Ord & Getis, 1995) to identify statistically significant spatial clusters of high mortality (hotspots) and low mortality (coldspots) for each time point. It then applies the non-parametric Mann–Kendall test (Kendall, 1975; Mann, 1945) to the time series of these clusters to classify each district into categories such as new, persistent, intensifying, diminishing, or sporadic, providing a dynamic view of mortality patterns.

A crucial step in our analysis was to stratify the data and conduct seperate analysis for Eastern European countries (Poland, Slovakia, and Czechia) and Western European countries (Belgium, Italy, France, Spain, Austria, and Germany). This stratification was essential because the systematically higher avoidable mortality levels in Eastern Europe would otherwise mask more nuanced, region-specific dynamics. By analysing the two groups separately, we could identify hot- and coldspots relative to their respective regional contexts, revealing patterns that would be obscured by the overarching East–West disparity. For completeness, an analysis of all countries combined is provided in Online Material 4, as well as a detailed description of the statistical method.

Based on the results of both the local Gi* and the Mann–Kendall test, the districts are being categorised to mirror their performance over time. This categorisation is predefined by the Environmental Systems Research Institute (ESRI, 2025) and is indicated in Table 1. From the standard 17-category output, we simplified the typology for clarity by combining the oscillating and sporadic categories into a single temporary group, as both represent inconsistent hot- or coldspot patterns over time. All calculations were conducted in R Core Team (2024), and we used the R package *sfdep* to conduct the emerging hotspot analysis (Parry & Locke, 2024).

**Table 1:** Categorisation of study area location based on the emerging hotspot analysis (Esri, 2025)

Pattern name	Definition
Intensifying hot or coldspot	A district that is a statistically significant hot or cold spot for at least 90% of the observation period, including the final time point. In addition, there is an overall increase in the intensity of clustering of low and high SDRs at each time point, and this increase is statistically significant
Persistent hot or coldspot	A statistically significant hot or cold spot in at least 90% of the observation period, with no significant trend in clustering intensity
Consecutive hot or coldspot	A district that is consistently identified as a hot or cold spot towards the final time points of the observation period, but was not a significant hot or cold spot before
New hot or coldspot	A statistically significant (p < 0.01) hot or cold spot in the final time point that is not statistically significant in the previous year under observation
Temporary hot or coldspot	Combines *sporadic* and *oscillating* patterns. It refers to a district identified as hot- or cold spots at the final time interval but have appeared irreguarly before
No pattern detected	The district does not fit into any defined hot or cold spot pattern

## Results

### Trends in Regional Amenable and Preventable

**Fig. 1:**
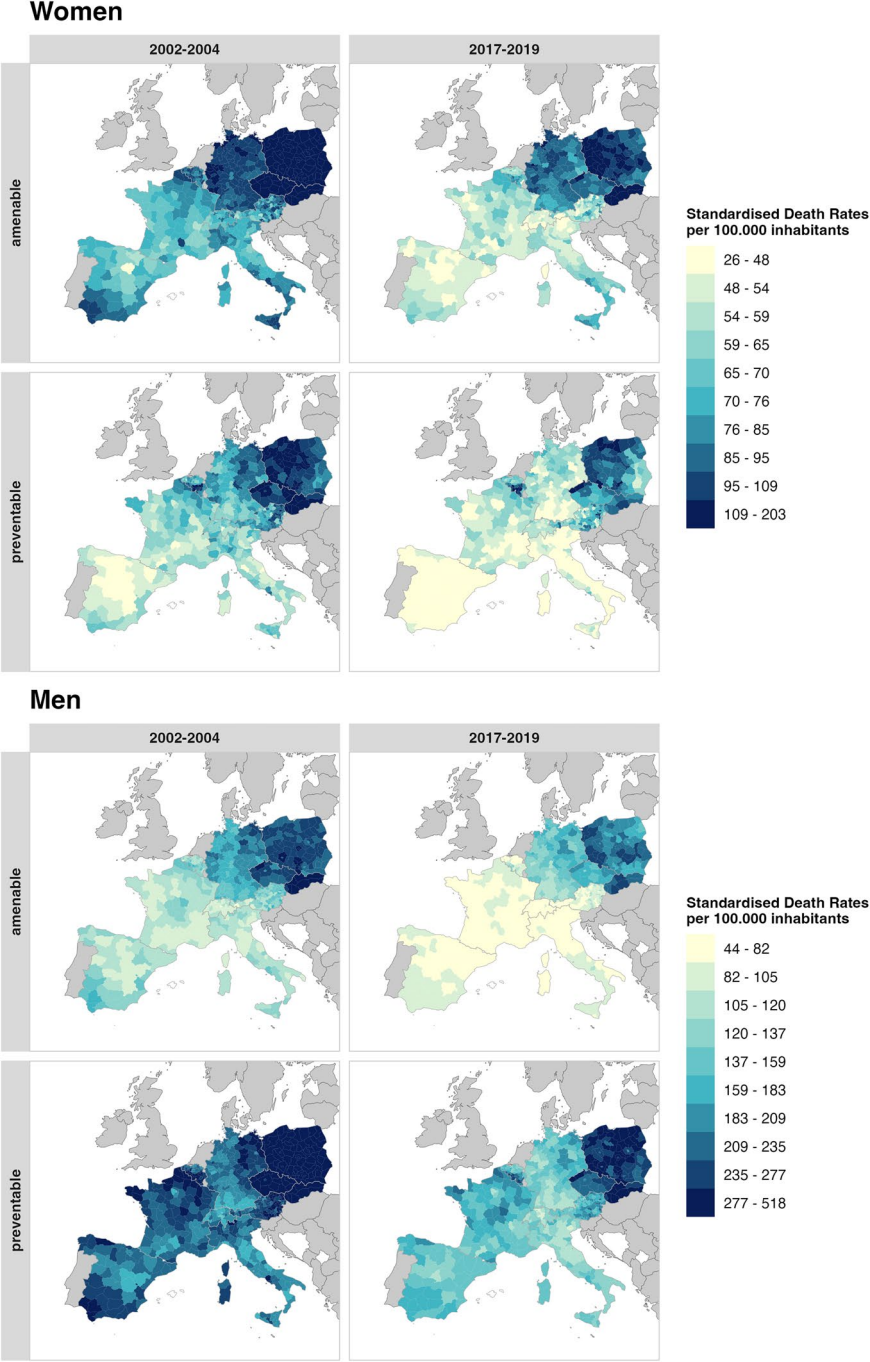
Standardised death rates from amenable and preventable causes (deaths per 100.000 inhabitants) in 2002–2004 and 2017–2019. The districts are being categorised by deciles for men and women, seperately

To showcase trends in avoidable mortality across all districts over time, we mapped SDR per 100,000 inhabitants for two time periods: 2002–2004 and 2017–2019, analysing both men and women separately, as well as distinguishing between amenable and preventable mortality, as depicted in Fig. 1. Overall, avoidable mortality rates have declined across most European countries; however, disparities persist between sexes, countries, regions, and across avoidable mortality classifications. Furthermore, we provided boxplots and interquartile ranges (IQRs) for more detailed information (see Online Resource 3).

#### Differences by Sex

Figure 1 reveals large disparities in amenable and preventable mortality within and across EU member states for both time points, also considering both sexes. Men have consistently higher SDRs than women, nearly double in most cases, across both periods and causes. The disparity between the sexes is more pronounced for preventable mortality than for amenable mortality. Women consistently show lower preventable SDRs compared to their amenable SDRs, while men tend to have higher preventable SDRs relative to their amenable SDRs. Across Europe, preventable mortality among men remains notably high, even though SDRs declined substantially between 2002–2004 and 2017–2019. Despite this improvement, men continue to record the highest SDRs overall, exceeding both amenable mortality and female rates. This is further reflected in the IQRs, which highlight considerable variation across the entire study region. In 2002–2004, preventable SDRs per 100,000 inhabitants ranged from an IQR of 29 among women to 82 among men, while in 2017–2019 the range extended from 24 among women to 62 among men. These values underline that preventable mortality, particularly among men, not only remains higher on average but also shows greater variability across member states. Additionally, IQRs for amenable mortality and for all countries are provided in Online Resource 3.

#### Leader and Laggards

Despite an overall decline in avoidable mortality across Europe, notable regional differences remain in the magnitude of these reductions. Albeit showing the largest reductions, Eastern European districts consistently exhibited the highest SDRs for both sexes. For amenable mortality, the Slovak regions bordering Hungary were among the districts with the highest SDRs in 2002–2004, whereas by 2017–2019, the highest SDRs were observed in Polish districts. Similarly, for preventable mortality, the highest rates were concentrated in Poland and, for females, in Czech districts bordering Germany. In contrast, the lowest amenable mortality was constantly found in Western and Eastern Europe. Male SDRs remained lowest in central and western Switzerland, adjacent Austrian regions and southern France. For females, the lowest SDRs were found in northern Spain, France, Switzerland and Austria. This geographic pattern, lower amenable SDRs in the west and south compared to the central eastern regions, remained stable over both periods. Germany, however, represents a notable exception to a simple east–west divide. It occupies an intermediate position, with amenable SDRs lower than its Eastern European neighbours but persistently higher than those in Western Europe.

#### Within-Country Variation

Notable mortality differences are also evident within national borders. In Spain and Italy, for instance, Southern regions have experienced more pronounced declines than their Northern counterparts, yet a persistent North–South gradient remains for both countries. A similar internal pattern is observed in Germany, where northeastern regions show higher amenable SDRs than those in the southwest. Notably, Germany stands apart from other older EU member states, as its internal disparities for amenable mortality have actually increased.

This widening gap in Germany contrasts with the general trend in countries like Spain, France, and Austria, which have made more progress in narrowing their regional divides. However, increases in regional inequality were not limited to Germany. For preventable mortality, disparities also rose for men in Poland and for women in Belgium. For amenable mortality, variation increased among women in Belgium and among men in Belgium, Poland, Czechia, and Italy. Throughout the study period, Switzerland consistently exhibited one of the lowest levels of within-country disparity.

#### Regional Patterns Spanning National Borders

Beyond national-level variations, several mortality patterns transcend borders, creating distinct transnational clusters. Some of these clusters represent contiguous high-mortality zones. For example, the high amenable SDRs observed in northern France are mirrored in the neighbouring Wallonia region of southern Belgium. This cross-border pattern is particularly pronounced for preventable mortality among women in Wallonia, which remained one of the worst-performing areas in 2017–2019.

Conversely, high-performing clusters also span national boundaries. The cross-border regions of Switzerland, western France, eastern Austria, and northern Italy collectively emerge as the best-performing area in the study, with only minor differences among them by 2017–2019.

Finally, some borders represent sharp discontinuities rather than shared patterns. The Polish and Czech districts adjacent to Germany, for instance, have high amenable SDRs and are among the worst-performing regions in their respective countries, but this trend does not extend across the border into Germany. While this sharp divide persists, there are signs of change; Eastern German regions, particularly those bordering Poland and Czechia, have experienced notable reductions in preventable SDRs, diminishing the historical east–west mortality gradient within Germany itself.

Overall, these spatial patterns suggest that borders have become more permeable among Western and Southern European countries, while a clear mortality divide remains between them and Eastern Europe, with Germany being an exception.

### Spatiotemporal Clusters

**Fig. 2:**
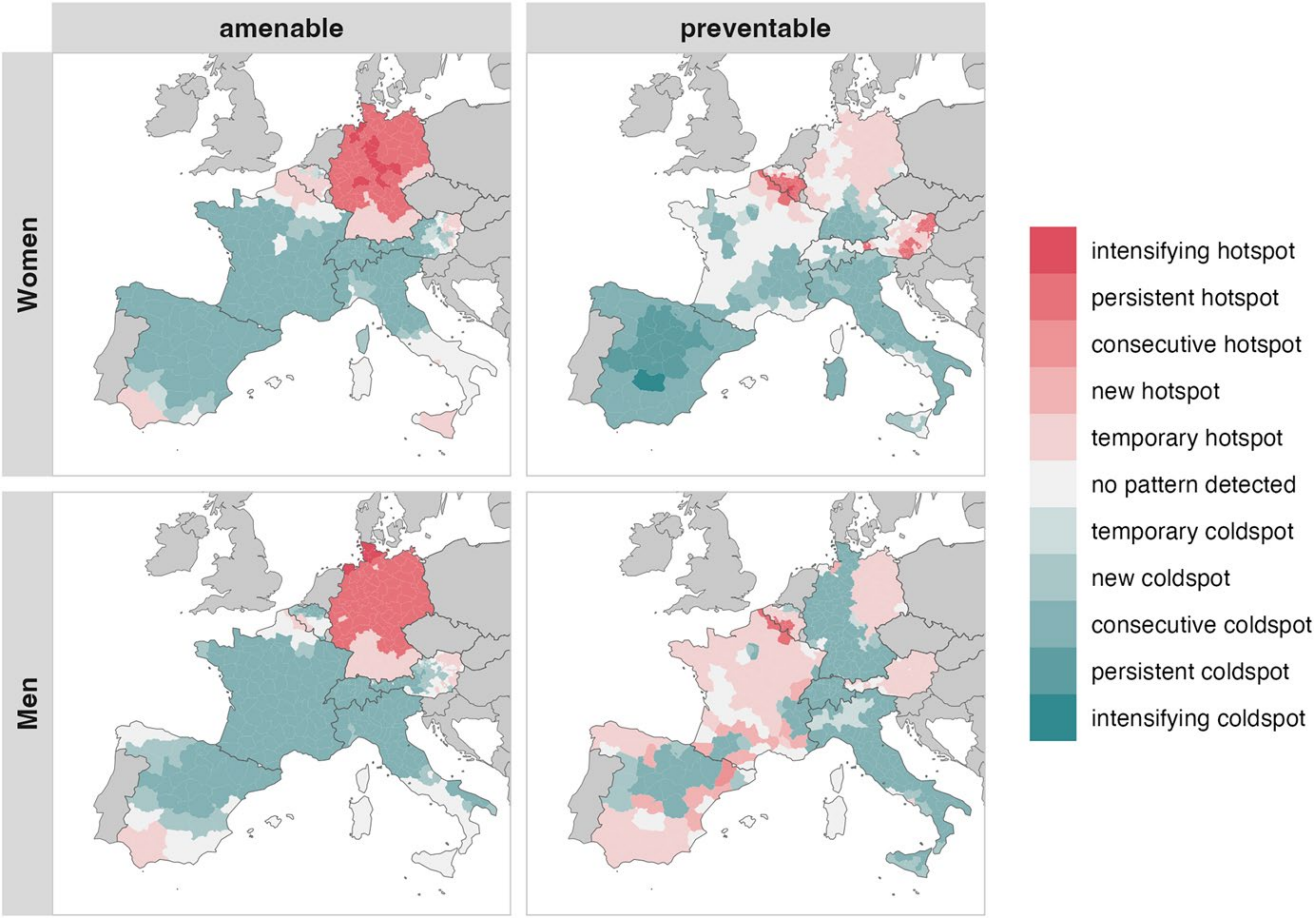
Spatiotemporal clusters of high and low mortality ratios of amenable and preventable causes by sex from 2002–2004 to 2017–2019 in Western European countries. Emerging hotspot calculations are based on standardised death rates

**Fig. 3:**
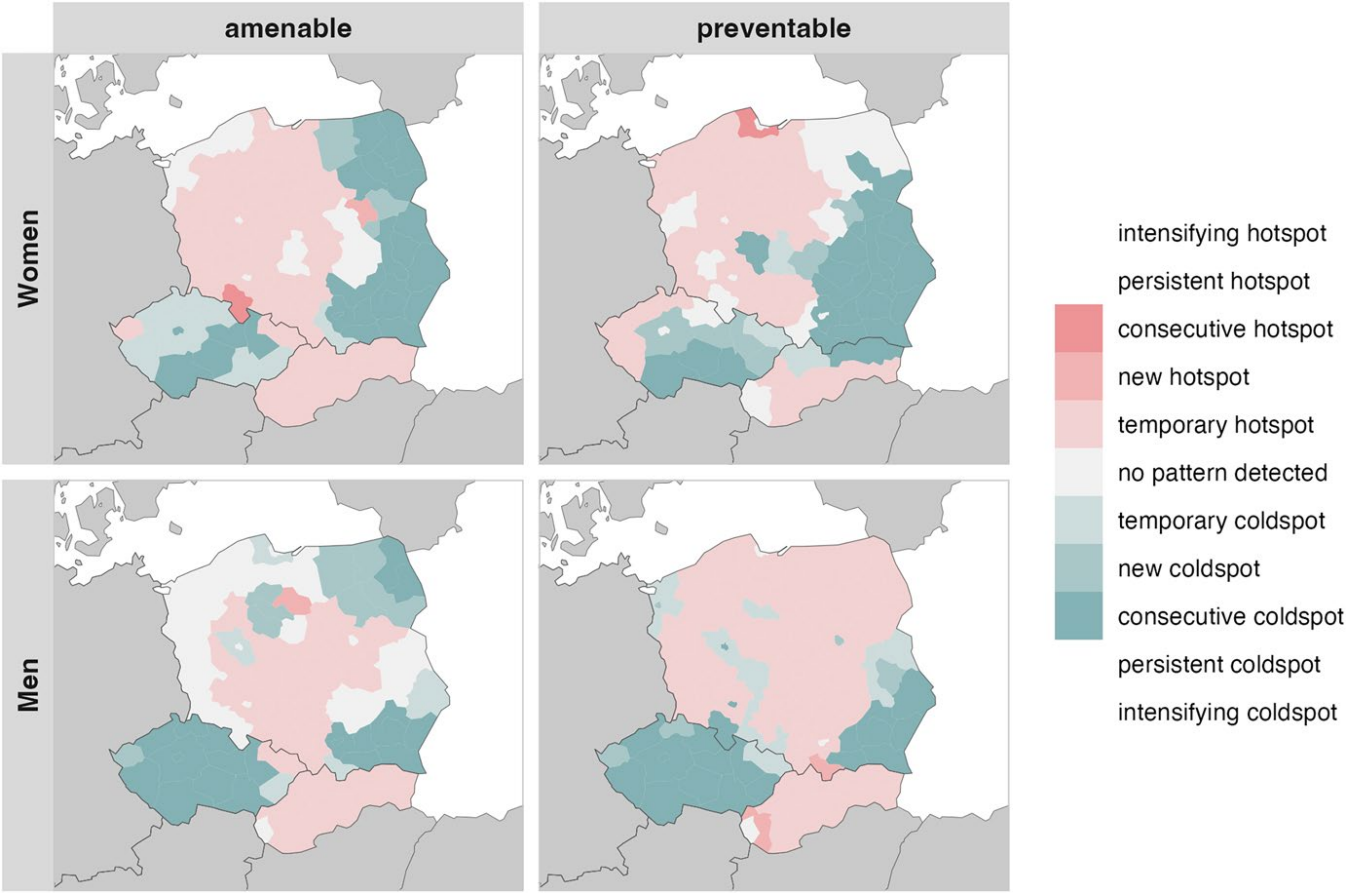
Spatiotemporal clusters of high and low mortality ratios of amenable and preventable causes by sex from 2002–2004 to 2017–2019 in Eastern European countries. Emerging hotspot calculations are based on standardised death rates from fig 1

To identify hot and coldspots of avoidable mortality, we summarised the results from the emerging hotspot analysis in Figs. 2 and 3. Figure 2 shows the spatiotemporal clusters across Western European countries, while Fig. 3 depicts the results for Eastern European countries.

#### Western European Countries

For amenable mortality, spatio-temporal clustering patterns among Western European countries are largely consistent across sexes. Persistent and intensifying hotspots, i.e. regions with higher mortality, are observed across northern and central Germany, while southern Germany is characterised by temporary hotspots. Additional temporary hotspots are identified in eastern Austrian districts bordering Eastern Europe, in southern Spain, and in southern Italy, among women. A cross-border cluster is detected in the French-Belgian border region; for women, this extends across the entire Wallonia region. Consecutive coldspots, i.e. regions with lower mortality, cover western Austria, northern Italy, Switzerland, and most of France and Spain, though among men the cluster is less extensive in Spain. Among men, consecutive coldspots are also found in the Flemish region of northern Belgium.

For preventable mortality, sex-specific differences are more pronounced. Among women, temporary hotspots are concentrated in eastern German districts and extend toward northwestern regions, with additional clusters across Austria, where some districts show persistent or consecutive clustering. Among men, temporary hotspots are more widespread, covering parts of France, Spain, and Belgium, with new clusters emerging in both France and Spain. As with amenable mortality, cross-border hotspots are detected along the French-Belgian border; however, they persist over time. For women, the cluster encompasses the entire Wallonia region, with one district bordering France identified as an intensifying hotspot. Coldspots for preventable mortality show distinct spatial structures. Among women, consecutive, persistent, and one intensifying coldspot are present across southern Germany, central and southern Switzerland, Spain, most of Italy, and southern France, forming a corridor from the Spanish to the Italian-Swiss borders. Among men, consecutive coldspots cover western and southern Germany, Switzerland, and most of Italy, with temporary coldspots in northern Italy and consecutive coldspots in southwestern France near the Italian and Swiss borders. Consecutive and newly emerging hotspots are additionally observed in north-central Spain and southern France near the Spanish border.

#### Eastern European Countries

Among Eastern European countries, neither intensifying nor persistent hot- or coldspots were found. Furthermore, only a single consecutive hotspot is located in a Polish district bordering Czechia among women, whereas no such cluster is detected among men. In contrast, consecutive coldspots are widespread. For women, these occur in central Czechia and eastern Poland, while for men they extend across nearly the entire Czechia, southeastern Poland, and several districts in the northeast. Temporary hotspots are more diffuse, covering much of western and central Poland, a few Czech districts, and the whole of Slovakia for women. For men, temporary hotspots are located in central and southern Poland, one Czech district, and most of Slovakia for men. Temporary coldspots are also observed, especially in many Czech regions and women. Overall, consecutive coldspots represent the dominant long-term clustering pattern for amenable mortality in both sexes, whereas hotspots are more short-lived and fragmented.

For preventable mortality, consecutive hotspots are again limited, with only a single Polish region among women and none among men. Instead, the most consistent patterns are consecutive coldspots, covering southern Czechia, eastern and southern Poland, and extending into northern Slovakia for women, while for men they encompass almost entire Czechia and southeastern Poland. Temporary hotspots are more widespread, spanning eastern and central Poland, Czech regions bordering Germany, and southern Slovakia for women, and nearly all of Poland and Slovakia (except Bratislava) for men. Temporary coldspots are relatively scattered in both sexes, with women showing clusters across Czechia and Slovakia, and men across Polish and Czech districts. Similar to amenable mortality, consecutive coldspots dominate the preventable mortality patterns in both sexes, though temporary hotspots are more extensive and dispersed.

As expected, conducting the emerging hotspot analysis across the entire study region (see Online Resource 4), yielded a somewhat different picture, particularly for Eastern European districts. While separate analyses identified consecutive coldspots within these countries, the combined analysis classifies large parts of Eastern Europe as persistent or even intensifying hotspots, except for Czech and western Polish districts, which appear as temporary hotspots. This pattern reflects the pronounced east–west gradient in Europe. Despite these shifts, Germany continues to display persistent hotspots for amenable mortality: in most eastern districts among men, and in northwestern districts among women, with some of these evolving into intensifying hotspots. Furthermore, the cross-border cluster of persistent hotspots along the French–Belgian border remains evident for women in preventable mortality, while among men the same cluster is visible but classified as temporary.

## Discussion

This study examined trends in amenable and preventable mortality across all districts in 10 European countries over almost two decades, and identified spatiotemporal clusters of leading (coldspots) and lagging (hotspots) regions. Our analysis revealed several key findings. First, while avoidable mortality rates have generally declined, marked disparities persist between sexes, avoidable mortality categories, and geographic regions. Second, although SDRs remain generally higher in Eastern Europe, our findings indicate that a simple East–West divide is challenged by Germany’s intermediate position for amenable mortality and the existence of persistent preventable mortality hotspots within Western Europe. Third, our spatiotemporal analysis revealed significant within-country differences alongside distinct transnational clusters that span national borders.

### The Persistence of Vanguards and Laggards

The overall reduction in avoidable mortality aligns with previous research, confirming that advances in healthcare and public health have contributed to declining mortality across Europe. (Hrzic & Vogt, 2024; Jarčuška et al., 2017; Mackenbach et al., 2015). However, our findings showed that, overall, Eastern European districts exhibit larger avoidable mortality rates compared to their Western European counterpart, confirming a general mortality divide between the two country blocks. The persistent differences in amenable mortality could be linked to structural differences in the healthcare systems. Eastern European countries typically face limitations in funding, higher out-of-pocket costs, and reduced access to primary care, which together heighten the risk of unmet medical needs (Kaminska & Wulfgramm, 2019; Popic & Schneider, 2018). In terms of preventable mortality, the east–west disparities are mainly driven by behavioural risk factors, such as alcohol and tobacco use, which are more prevalent in Eastern European populations (Santucci et al., 2022; Trias-Llimós et al., 2018).

However, our district-level analysis reveals that this broad East–West dichotomy is not absolute and masks crucial underlying dynamics. Germany, in particular, disrupts a simple divide, acting as an intermediate case. Its persistently high amenable mortality rates align with literature identifying Germany as a "taillight" in life expectancy among high-income countries, largely due to sustained excess mortality from cardiovascular diseases (Jasilionis et al., 2023). Furthermore, analysing Eastern and Western European country blocks separately, our study identified significant positive developments, such as consecutive coldspots in Czechia and Western Poland. This stratified view also revealed concerning trends of stagnation in the West, evidenced by persistent hotspots for amenable mortality within Germany and preventable mortality in Wallonia, Belgium. This finding of simultaneous progress and stagnation within Western European districts resonates with life expectancy trends, which similarly show a complex pattern of catching-up in some areas and faltering progress in others (Stroisch et al., 2025b). This underscores that convergence is not uniform and that both vanguard and laggard dynamics are occurring simultaneously across the continent.

### Beyond National Averages

The emergence of these distinct vanguard and laggard dynamics within European countries highlights that health outcomes are often shaped more by regional context than by national averages alone. This is first evident in the entrenched disparities within countries. In Spain, for example, mortality excess in Western Andalusia persisted, largely driven by high cancer-related deaths in older age groups (Ocaña-Riola & Mayoral-Cortés, 2010; Santos-Sánchez et al., 2020). In Poland, regional inequalities in avoidable mortality were associated with the availability of primary care and specialist physicians, highlighting the role of local healthcare infrastructure in shaping outcomes (Sagan et al., 2022; Wróblewska, 2017).

While such inner-country differences are known in the literature, we find that these high avoidable mortality regions do not stop at the border. We revealed a persistent cross-border hotspot along the French-Belgian border for preventable mortality and a temporary hotspot for amenable mortality. This cluster was consistent for both sexes and also when analysing Western and Eastern European countries together. The consistent underperformance of this region reflects shared structural vulnerabilities, such as deindustrialisation, demographic decline, socioeconomic disadvantage and unequal healthcare access, that affect both sides of the border (Bonnet & d’Albis, 2020; Eggerickx et al., 2018; Otavova et al., 2024). The persistence of these clusters points not only to national-level healthcare system limitations but also to a broader, transnational inability to reduce mortality in structurally disadvantaged, lagging areas.

In contrast, we identified a large and stable cross-border coldspot, encompassing many districts of southern Germany (preventable), western Austria (amenable), Italy, Switzerland, Southern France, and Spain. These areas consistently demonstrate some of the lowest amenable or preventable mortality rates in Europe. Their success reflects a shared context of long-term economic prosperity and high standards of healthcare access, creating a transnational region of vanguards whose performance transcends national healthcare system models.

Overall, these spatial patterns indicate that national borders are not always the most meaningful lines when it comes to explaining health outcomes. Instead, local socioeconomic and structural determinants of health play a central role, spilling also across administrative boundaries.

### Strengths and Limitations

While this study provides valuable insights into the spatiotemporal dynamics of avoidable mortality in Europe, it is not free from limitations. Previous scholars have noted shortcomings of the concept of avoidable mortality, such as age restrictions and the selection of causes of death. Furthermore, variations in death certification practices across different countries complicate cross-national comparisons (Hrzic et al., 2025; Mackenbach et al., 2015; Mühlichen et al., 2023). To mitigate potential inconsistencies in coding precision, we classified cause-specific death groups based on 3-digit ICD codes, rather than using the more granular 4-digit codes. Despite its limitations, avoidable mortality, when analysed alongside all-cause mortality, continues to offer valuable insights and remains an essential tool for comparative health systems research.

Moreover, working with district-level data brings both strengths and constraints: while it enables the detection of local patterns, small population sizes can introduce statistical noise. Aggregating data into three-year periods and using emerging hotspot analysis helped address these concerns by emphasising persistent over transient trends.

Another limitation lies in the scope of country selection. While the study includes diverse healthcare systems from across Europe, data availability constrained inclusion. Facilitating access to granular regional data across more EU countries would greatly enhance future research and provide a more comprehensive understanding of avoidable mortality patterns at the European level.

Despite these limitations, this study represents the first effort to examine avoidable mortality at the district level across both old and new EU member states. Additionally, by utilising district-level data, we were able to uncover variations not only between individual EU member states but also within them. This approach enabled us to identify, among others, cross-border clusters of avoidable mortality, thereby highlighting spatial health inequalities that transcend national boundaries.

### Conclusion & Policy Implications

This study demonstrated that despite overall progress, significant and geographically concentrated inequalities in avoidable mortality persist across and within the national borders of European countries. Our findings challenge the EU’s ambition to harmonise health standards and foster convergence, suggesting that health disparities are shaped by a complex interplay of national, regional, and socioeconomic factors that current policies cannot fully address. Persistent variations in avoidable mortality across and within European member states, including cross-border regions of high and low mortality, indicate that health outcomes cannot be explained by national health systems alone.

The limitations of broad, national-level interventions are particularly evident in entrenched cross-border hotspots, such as the one observed at the French-Belgian border. These lagging regions require targeted, place-based strategies and strong cross-border collaborations addressing shared structural determinants of health. Conversely, the emergence of a large, prosperous coldspot provides a powerful model of a transnational area with low mortality. This successful region spans Western Austria, Southern Germany, Switzerland, Northern and Central Italy, South-Western France, and North-Eastern Spain. Frameworks like the EU’s Open Method of Coordination could be leveraged more effectively to study and disseminate best practices from these vanguard regions, fostering mutual learning across borders.

While EU cohesion policy has channelled important resources into health infrastructure in disadvantaged regions, its impact is limited by reliance on soft governance. These instruments often lack binding authority to enforce convergence and, as Van Der Zanden et al., (2024) note, tend to prioritise acute cross-border threats over long-standing socioeconomic determinants of health. This, together with a deficit in a shared “European mindset” for public health, hinders the development of a comprehensive strategy capable of producing systemic and lasting change (Van Der Zanden et al., 2024, p.11).

Our findings highlight the value of a more regionally sensitive and cohesive approach to EU policies. Rather than focusing solely on national frameworks, policy should integrate health equity objectives more deeply into broader regional development and cohesion strategies. This involves ensuring that investments in infrastructure and economic growth also produce measurable public health improvements. By strategically promoting and funding transnational, region-based initiatives, both to address disparities in lagging regions and to disseminate best practices from leading regions, the EU is uniquely positioned to stimulate the place-based innovations and partnerships needed to pursue health convergence across its diverse territories.

## Supplementary Information

Below is the link to the electronic supplementary material.

Supplementary file1 (DOCX 24 KB)

Supplementary file2 (CSV 411 KB)

Supplementary file3 (DOCX 467 KB)

Supplementary file4 (DOCX 566 KB)

## Data Availability

The data and R code for this study are available on request from the corresponding author.
