# Supplementary material for: Spatial Differences in Avoidable Mortality Across 581 European Districts, 2002–2019
Source: Eur J Popul. 2025 Dec 9;42(1):5. doi: 10.1007/s10680-025-09761-7 (PMC12791106; doi:10.1007/s10680-025-09761-7)
Supplement: Supplementary file 1 — Supplementary file1 (DOCX 24 KB) [file 10680_2025_9761_MOESM1_ESM.docx]

Online Resource 1 - Classification of Avoidable Mortality

**Article name:** Spatial Differences in Avoidable Mortality Across 581 European Districts, 2002-2019

**Journal name:** European Journal of Populaiton

**Author names:** Sophie Stroisch^1,2^, Michael Mühlichen^3^, Pavel Grigoriev^3^, Tobias Vogt^1,4^

**Affiliations:**

1 Population Research Centre, Faculty of Spatial Sciences, University of Groningen, The Netherlands

2 Institute of Social Sciences, Carl von Ossietzky University of Oldenburg, Germany

3 Federal Institute for Population Research (BiB) Wiesbaden, Germany

4 Prasanna School of Public Health, Manipal Academy of Higher Education, Manipal, Karnataka, India

**Correspondence:** Sophie Stroisch, [s.stroisch@rug.nl](mailto:s.stroisch@rug.nl)

Causes considered **amenable** to health care, ages 0-74 (unless otherwise stated), according to Mühlichen et al. (2023)

| **Disease group** | **Cause of death** | **Age** | **ICD-8 code** | **ICD-9 code** | **ICD-10 code** |
| --- | --- | --- | --- | --- | --- |
| *Infectious* | Intestinal infections | 0-14 | 000-9 | 001-9 | A00-9 |
|  | Tuberculosis |  | 010-9 | 010-8, 137 | A15-9, B90 |
|  | Whooping cough | 0-14 | 033 | 033 | A37 |
|  | Measles | 1-14 | 055 | 055 | B05 |
|  | Other infections |  | 032, 034-8, 040-3, 084, 381-3, 681-2, 720 | 032, 034-8, 045, 084,  381-3, 681-2, 730 | A35-6, A38-41, A46, A80, B50-4, H65-70, L03, M86, M89-90 |
| *Neoplasm* | Colorectal cancer |  | 153-4 | 153-4 | C18-21 |
|  | Bone cancer |  | 170 | 170 | C40-1 |
|  | Skin cancer |  | 172-3 | 172-3 | C43-4 |
|  | Female breast cancer |  | 174 | 174 | C50 |
|  | Cervical cancer |  | 180 | 180 | C53 |
|  | Uterine cancer |  | 182 | 179, 182 | C54, C55 |
|  | Prostate cancer |  | 185 | 185 | C61 |
|  | Testicular cancer |  | 186 | 186 | C62 |
|  | Bladder cancer |  | 188 | 188 | C67 |
|  | Eye cancer |  | 190 | 190 | C69 |
|  | Thyroid cancer |  | 193 | 193 | C73 |
|  | Hodgkin’s lymphoma |  | 201 | 201 | C81 |
|  | Leukaemia | 0-44 | 204-7 | 204-8 | C91-5 |
|  | Benign neoplasm |  | 210-28 | 210-29 | D10-36 |
| *Endocrine* | Diseases of the thyroid |  | 240-6 | 240-6 | E00-7 |
|  | Diabetes mellitus (50%) | 0-49 | 250 | 250 | E10-4 |
| *Neurological* | Bacterial meningitis |  | 320 | 320 | G00 |
|  | Epilepsy |  | 345 | 345 | G40-1 |
| *Cardiovascular* | Rheumatic heart disease |  | 390-8 | 390-8 | I00-9 |
|  | Hypertensive disease |  | 400-4 | 401-5 | I10-3, I15 |
|  | Ischaemic heart disease (50%) |  | 410-4 | 410-4 | I20-5 |
|  | Heart failure |  | 427-9 | 428-9 | I50-1 |
|  | Cerebrovascular disease (50%) |  | 430-8 | 430-8 | I60-9 |
| *Respiratory* | Influenza |  | 470-4 | 487-8 | J09-11 |
|  | Pneumonia |  | 480-6 | 480-6 | J12-8 |
|  | Asthma | 0-44 | 493 | 493 | J45-6 |
|  | Other respiratory diseases | 1-14 | 460-6, 500-519 | 460-79, 494-495, 497-519 | J00-8, J20-39, J47-99 |
| *Digestive* | Peptic ulcer |  | 531-4 | 531-4 | K25-8 |
|  | Appendicitis |  | 540-3 | 540-3 | K35-8 |
|  | Abdominal hernia |  | 550-3 | 550-3 | K40-6 |
|  | Cholelithiasis and cholecystitis |  | 574-6 | 574-5 | K80-2 |
| *Genitourinary* | Nephritis and nephrosis |  | 580-4, 792 | 580-9 | N00-7, N17-9, N25-7 |
|  | Hyperplasia of prostate |  | 600 | 600 | N40 |
| *Maternal/infant* | Maternal death |  | 630-78 | 630-79 | O00-99 |
|  | Perinatal deaths (excl.   stillbirths) |  | 760-79 | 760-79 | P00-96, A33 |
|  | Congenital anomalies |  | 740-59 | 740-59 | Q00-99 |
| *External* | Treatment complications |  | E930-6 | E870-9 | Y60-84 |

Causes considered **preventable** through primary prevention; ages 0-74 (unless otherwise stated), according to Mühlichen et al. (2023)

| **Disease group** | **Cause of death** | **Age** | **ICD-8 code** | **ICD-9 code** | **ICD-10 code** |
| --- | --- | --- | --- | --- | --- |
| *Infectious* | Hepatitis |  | 070 | 070 | B15-9 |
|  | HIV/AIDS |  | 137 | 042-4 | B20-4 |
|  | Sexually transmitted diseases |  | 090-9 | 090-9 | A50-64 |
| *Neoplasm* | Cancer of lip, oral cavity,   pharynx |  | 140-9 | 140-9 | C00-14 |
|  | Cancer of oesophagus |  | 150 | 150 | C15 |
|  | Cancer of stomach |  | 151 | 151 | C16 |
|  | Cancer of liver |  | 155 | 155 | C22 |
|  | Cancer of larynx |  | 161 | 161 | C32 |
|  | Cancer of trachea, bronchus,   lung |  | 162 | 162 | C33-4 |
| *Endocrine/* | Nutritional deficiency anaemia |  | 280-1 | 280-1 | D50-3 |
| *nutritional* | Diabetes mellitus (50%) | 0-49 | 250 | 250 | E10-4 |
|  | Alcohol and drug related   diseases |  | 291, 303-4 | 291-2, 303-5 | F10-6, F18-9 |
| *Cardiovascular* | Ischaemic heart disease (50%) |  | 410-4 | 410-4 | I20-5 |
|  | Cerebrovascular disease (50%) |  | 430-8 | 430-8 | I60-9 |
|  | Aortic aneurysm |  | 441 | 441 | I71 |
| *Respiratory* | Chronic obstructive pulmonary  disease |  | 490-2 | 490-2, 496 | J40-4 |
| *Digestive* | Cirrhosis of liver |  | 571 | 571 | K70, K73-4 |
| *External* | Land transport accidents |  | E810-27 | E810-29, E846-8 | V01-4, V06, V09-80, V82-9, V98-9 |
|  | Falls |  | E880-6 | E880-6, E888 | W00-19 |
|  | Fires, burns |  | E890-9 | E890-9 | X00-9 |
|  | Accidental poisonings |  | E850-77 | E850-69 | X40-9 |
|  | Drowning |  | E910 | E910 | W65-74 |
|  | Suicide and self-inflicted   injuries |  | E950-9, E980-9 | E950-9, E980-9 | X60-84, Y10-34 |
|  | Violence |  | E960-9 | E960-9 | X85-Y09 |
