# Supplementary material for: Spatial Differences in Avoidable Mortality Across 581 European Districts, 2002–2019
Source: Eur J Popul. 2025 Dec 9;42(1):5. doi: 10.1007/s10680-025-09761-7 (PMC12791106; doi:10.1007/s10680-025-09761-7)
Supplement: Supplementary file 3 — Supplementary file3 (DOCX 467 KB) [file 10680_2025_9761_MOESM3_ESM.docx]

Online Resource 3 – Boxplots and Interquartile Ranges

**Article name:** Spatial Differences in Avoidable Mortality Across 581 European Districts, 2002-2019

**Journal name:** European Journal of Population

**Author names:** Sophie Stroisch^1,2^, Michael Mühlichen^3^, Pavel Grigoriev^3^, Tobias Vogt^1,4^

**Affiliations:**

1 Population Research Centre, Faculty of Spatial Sciences, University of Groningen, The Netherlands

2 Institute of Social Sciences, Carl von Ossietzky University of Oldenburg, Germany

3 Federal Institute for Population Research (BiB) Wiesbaden, Germany

4 Prasanna School of Public Health, Manipal Academy of Higher Education, Manipal, Karnataka, India

**Correspondence:** Sophie Stroisch, [s.stroisch@rug.nl](mailto:s.stroisch@rug.nl)

**Interquartile Ranges**

|  |  | **Women** | |  | **Men** | |
| --- | --- | --- | --- | --- | --- | --- |
| **Category** | **Country** | **2002-2004** | **2017-2019** |  | **2002-2004** | **2017-2019** |
| amenable | Austria | 19.6 | 13 |  | 30.3 | 19.2 |
|  | Belgium | 12.1 | 16.4 |  | 20.7 | 28.5 |
|  | Switzerland | 11.3 | 5.5 |  | 16.8 | 11.7 |
|  | Germany | 11 | 14.7 |  | 28.4 | 32.8 |
|  | Spain | 12.9 | 8.3 |  | 19.1 | 14 |
|  | France | 7.6 | 8.3 |  | 18.9 | 13.5 |
|  | Italy | 11.4 | 10.3 |  | 14 | 17.5 |
|  | Czechia | 21.3 | 11.3 |  | 18 | 23 |
|  | Poland | 18.3 | 15.5 |  | 32.4 | 33.1 |
|  | Slovakia | 23.2 | 9.1 |  | 36.8 | 23.2 |
|  | East^1^ | 22.8 | 22.7 |  | 37.4 | 40.8 |
|  | West^2^ | 24 | 20.7 |  | 43.6 | 33.2 |
|  | All | 31.3 | 32.4 |  | 69.9 | 63.6 |
|  |  |  |  |  |  |  |
| preventable | Austria | 19.1 | 18.6 |  | 52.6 | 45.9 |
|  | Belgium | 19.1 | 22.8 |  | 71.7 | 60.6 |
|  | Switzerland | 8.7 | 8.9 |  | 31.4 | 23.4 |
|  | Germany | 15.6 | 12.5 |  | 42.9 | 31.4 |
|  | Spain | 8.8 | 6.2 |  | 41 | 17.2 |
|  | France | 11.5 | 10.8 |  | 37.5 | 31.3 |
|  | Italy | 10 | 7.3 |  | 25.7 | 19.9 |
|  | Czechia | 21.6 | 17 |  | 58.8 | 28.5 |
|  | Poland | 22.3 | 20.7 |  | 52.3 | 58.2 |
|  | Slovakia | 16.6 | 11.9 |  | 67.9 | 24.6 |
|  | East | 20.6 | 20 |  | 66.7 | 55.7 |
|  | West | 21.2 | 18 |  | 55 | 40.1 |
|  | All | 28.9 | 24 |  | 81.8 | 62.1 |

^1^ Poland, Czechia, Slovakia

^2^ Austria, Belgium, Germany, Switzerland, France, Italy, Spain

**Boxplots**

**
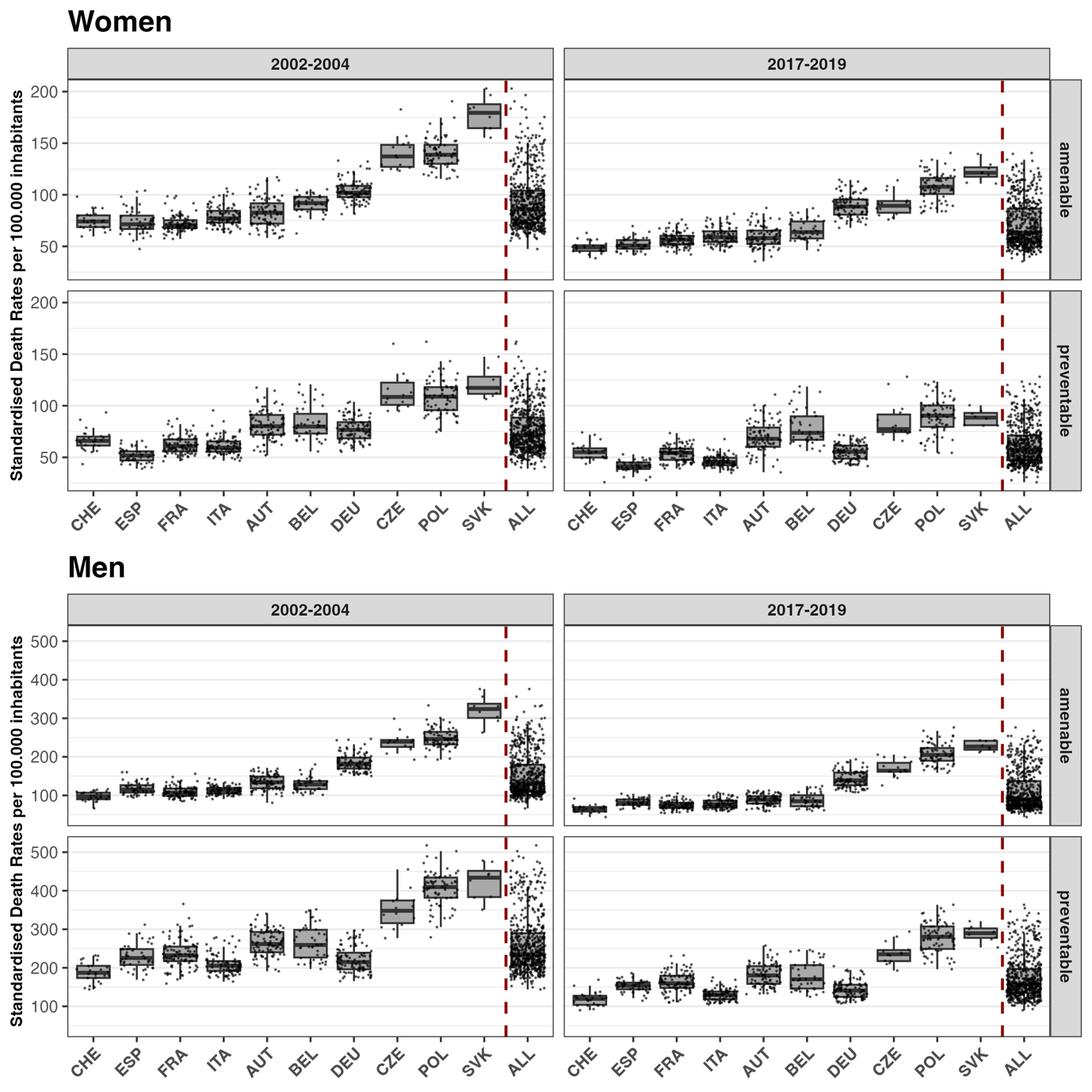
**
