# Supplementary material for: Spatial Differences in Avoidable Mortality Across 581 European Districts, 2002–2019
Source: Eur J Popul. 2025 Dec 9;42(1):5. doi: 10.1007/s10680-025-09761-7 (PMC12791106; doi:10.1007/s10680-025-09761-7)
Supplement: Supplementary file 4 — Supplementary file4 (DOCX 566 KB) [file 10680_2025_9761_MOESM4_ESM.docx]

Online Resource 4 – Supplementary Material to the Emerging Hotspot Analysis

**Article name:** Spatial Differences in Avoidable Mortality Across 581 European Districts, 2002-2019

**Journal name:** European Journal of Populaiton

**Author names:** Sophie Stroisch^1,2^, Michael Mühlichen^3^, Pavel Grigoriev^3^, Tobias Vogt^1,4^

**Affiliations:**

1 Population Research Centre, Faculty of Spatial Sciences, University of Groningen, The Netherlands

2 Institute of Social Sciences, Carl von Ossietzky University of Oldenburg, Germany

3 Federal Institute for Population Research (BiB) Wiesbaden, Germany

4 Prasanna School of Public Health, Manipal Academy of Higher Education, Manipal, Karnataka, India

**Correspondence:** Sophie Stroisch, [s.stroisch@rug.nl](mailto:s.stroisch@rug.nl)

**Emerging Hotspot Analysis – Whole Study Area**


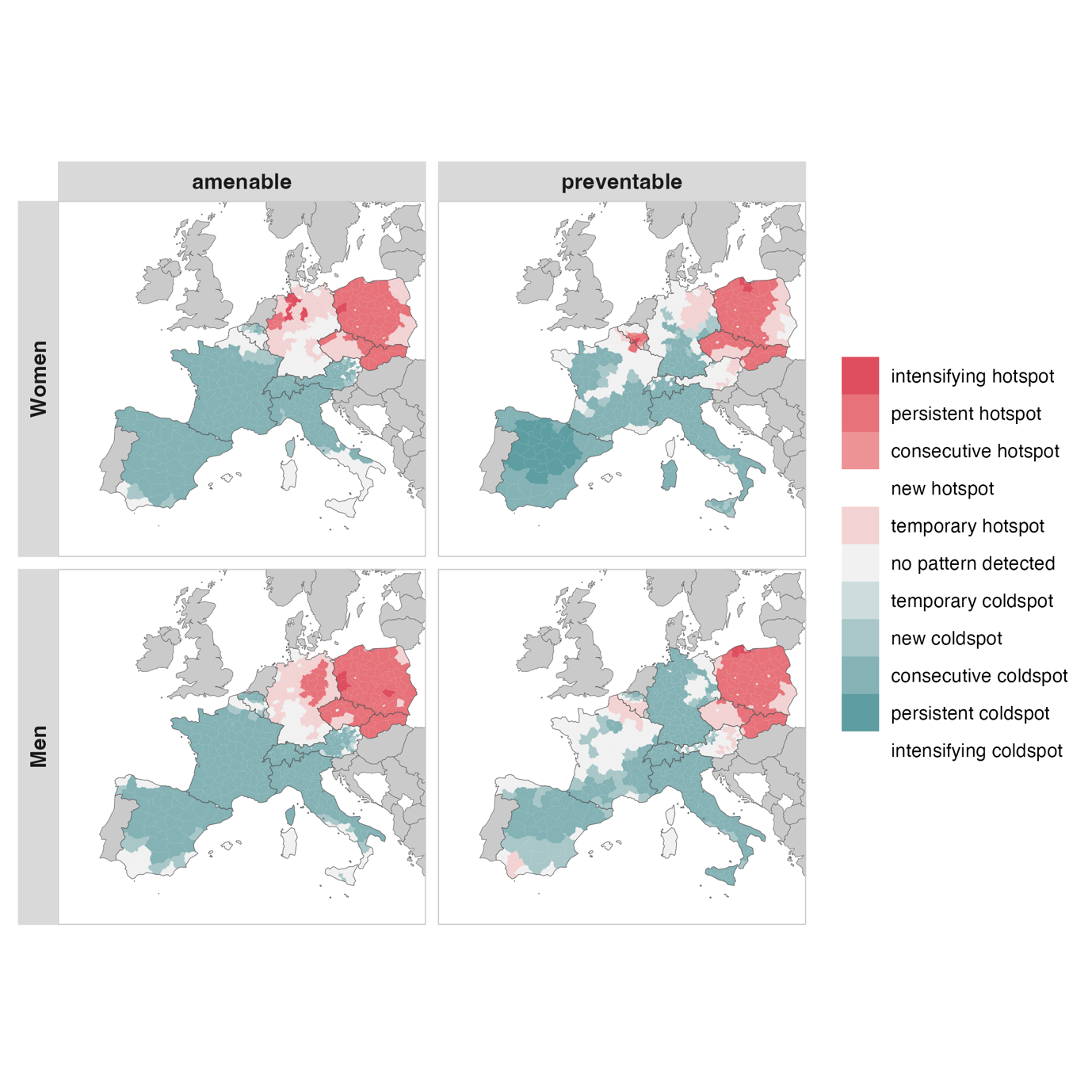
Spatiotemporal clusters of high and low mortality ratios of amenable and preventable causes by sex from 2002-2004 to 2017-2019 in Western European

**Method**

Emerging Hotspot Analysis combines spatial hotspot detection using the Getis-Ord Gi* statistic (Ord & Getis, 1995) with temporal trend detection using the non-parametric Mann-Kendall test (Kendall, 1975; Mann, 1945). The Getis-Ord Gi* statistic is a local spatial statistic that identifies clusters of unusually high or low values, in our case, high or low SDRs, based on the values in neighbouring districts. A queen contiguity spatial weights matrix was applied, meaning regions are considered neighbours if they share a border or a vertex. For each district and at each time point within our study period, the Gi* statistic is calculated by comparing the local sum of SDRs across a district and its neighbours to the global sum across all districts. This results in a z-score for each district at each time point. A high positive z-score indicates that the district and its neighbours are part of a statistically significant spatial cluster with elevated SDRs (a “hotspot”), while a high negative z-score indicates a statistically significant cluster with low SDRs (a “coldspot”). The null hypothesis of the local Gi* test is that there is no spatial clustering and that the observed pattern of SDRs is randomly distributed across space. The threshold was set to 0.01, meaning that only spatial clusters identified within 99%-confidence intervals were considered.

To evaluate whether these local clusters are persistent, new, or intensifying over time, the emerging hotspot analysis applies the Mann-Kendall trend test to the Gi* z-score time series of each district. The Mann-Kendall test identifies whether there is a statistically significant monotonic trend, either increasing or decreasing, over the entire study period. The output is Kendall’s tau, a value between -1 and +1, where +1 indicates a strong increasing trend, -1 a strong decreasing trend, and 0 no trend. By combining spatial clustering and temporal trend analysis, the Emerging Hotspot Analysis allows us to identify each district into hotspot categories such as new, persistent, or intensifying, thus providing a spatiotemporal profile of preventable and amenable mortality trends across Europe, separately for men and women.

Based on the results of both the local Gi* and the Mann-Kendall test, the districts are being categorised mirroring their performance over time. This categorisation is predefined by the Environmental Systems Research Institute (ESRI, 2025) and is indicated in Table 1 in the manuscript. In total, the typology consists of 17 categories, including *new, consecutive, persistent, intensifying, diminishing, sporadic, oscillating*, and *historic* hot- and coldspots, as well as *no pattern detected*. In our analyses, the categories *historic* and *diminishing* did not occur. Furthermore, we combined *oscillating* and *sporadic* into one group named *temporary*, as both indicate that a district was a hot- or coldspot at certain time points but not consistently over the study period. The distinction between them lies in the fact that oscillating districts switch between hot- and coldspot status, whereas sporadic districts are identified as only one or the other at isolated time points.
